# Chronic nicotine impairs sparse motor learning via striatal fast‐spiking parvalbumin interneurons

**DOI:** 10.1111/adb.12956

**Published:** 2020-08-06

**Authors:** Baeksun Kim, Heh‐In Im

**Affiliations:** ^1^ Convergence Research Center for Diagnosis, Treatment and Care System of Dementia (DTC) Korea Institute of Science and Technology (KIST) Seoul Republic of Korea; ^2^ Division of Bio‐Medical Science and Technology, KIST School Korea University of Science and Technology (UST) Seoul Republic of Korea; ^3^ Center for Neuroscience Korea Institute of Science and Technology (KIST) Seoul Republic of Korea

**Keywords:** dorsal striatum, extracellular single‐unit recording, fast‐spiking interneurons, local field potential, motor learning, nicotine withdrawal

## Abstract

Nicotine can diversely affect neural activity and motor learning in animals. However, the impact of chronic nicotine on striatal activity in vivo and motor learning at long‐term sparse timescale remains unknown. Here, we demonstrate that chronic nicotine persistently suppresses the activity of striatal fast‐spiking parvalbumin interneurons, which mediate nicotine‐induced deficit in sparse motor learning. Six weeks of longitudinal in vivo single‐unit recording revealed that mice show reduced activity of fast‐spiking interneurons in the dorsal striatum during chronic nicotine exposure and withdrawal. The reduced firing of fast‐spiking interneurons was accompanied by spike broadening, diminished striatal delta oscillation power, and reduced sample entropy in local field potential. In addition, chronic nicotine withdrawal impaired motor learning with a weekly sparse training regimen but did not affect general locomotion and anxiety‐like behavior. Lastly, the excitatory DREADD hM3Dq‐mediated activation of striatal fast‐spiking parvalbumin interneurons reversed the chronic nicotine withdrawal‐induced deficit in sparse motor learning. Taken together, we identified that chronic nicotine withdrawal impairs sparse motor learning via disruption of activity in striatal fast‐spiking parvalbumin interneurons. These findings suggest that sparse motor learning paradigm can reveal the subtle effect of nicotine withdrawal on motor function and that striatal fast‐spiking parvalbumin interneurons are a neural substrate of nicotine's effect on motor learning.

## Graphical abstract

Chronic nicotine diminished the activity of fast‐spiking parvalbumin interneurons and degrades delta oscillation power as well as signal complexity of local field potentials in the dorsal striatum. Additionally, chronic nicotine impaired sparse motor skill learning specifically during nicotine withdrawal. Restoring the activity of striatal fast‐spiking parvalbumin interneurons rescued the chronic nicotine‐induced deficit in sparse motor skill learning.

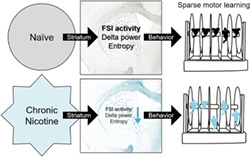

## 1 INTRODUCTION

Tobacco addiction is the main cause of preventable death worldwide.^1^ Understanding the behavioral consequences of nicotine and the neural mechanism thereof are critical to treatment development. A core characteristic of nicotine is its diverse and complex impact on behavior,^2^, ^3^, ^4^ which suggests the necessity for fine examination of nicotine's effect on phenotype. Recently, nicotine has been shown to control a variety of motor functions, including motor activity, motor stereotypy, and motor learning.^4^, ^6^ Interestingly though, chronic nicotine did not affect motor learning with a daily training regimen,^7^, ^8^ suggesting that nicotine‐induced motor learning deficit may be a subtle phenotype that should be revealed through a unique behavioral paradigm. Moreover, little is known about the neural mechanism underlying the potential effect of chronic nicotine on motor function.

The dorsal striatum is the major output of basal ganglia and is deeply involved in adaptive motor control.^9^, ^10^ Aberrant neural processing within the dorsal striatum can impair motor learning as observed in movement disorders.^11^ Previous studies have found that chronic nicotine can disrupt neuronal activity within the dorsal striatum ex vivo,^12^, ^13^ suggesting that nicotine‐dependent alteration of motor behavior could require striatal activity. However, the in vivo influence of chronic nicotine on the neural activity within the dorsal striatum and the potential role that striatal neurons play in nicotine‐dependent alteration of motor learning remains elusive.

In this study, we adopted 6 weeks of longitudinal single‐unit recording and a sparse training regimen on rotarod to study the impact of chronic nicotine exposure and withdrawal on the striatal neural activity in mice. Specifically, we compared the in vivo activity of striatal medium spiny neurons (MSNs) and fast‐spiking interneurons (FSIs) before, during, and after chronic nicotine treatment. MSNs comprise ~95% of the striatal neuron population and are the output neurons of the striatum,^14^ whereas FSIs are the main source of GABAergic feedforward inhibition in the striatum and marked by parvalbumin immunoreactivity.^15^, ^16^, ^17^

Subsequently, we applied the chemogenetic approach to selectively modulate the activity of striatal fast‐spiking parvalbumin interneurons to examine their role in nicotine‐dependent alteration of motor learning. Here, we applied a sparse motor learning paradigm. The sparse training regimen renders the behavioral task more demanding, which has been shown to be more sensitive to subtle deficit in behavior.^18^, ^19^, ^20^ Furthermore, sparse training is a valid means of enhancing motor skill since the retention of motor learning can last for more than 7 days.^21^, ^22^

## 2 MATERIALS AND METHODS

### 2.1 Subjects

For in vivo single‐unit recording, 3‐month‐old C57BL/6J male mice (KIST Research Animal Resource Center, Seoul, Republic of Korea) were single‐housed and extensively handled for more than 2 weeks before tetrode microdrive implantation surgery. For behavioral experiments, 2‐ to 3‐month‐old C57BL/6J male mice (KIST Research Animal Resource Center) or B6.129P2‐Pvalb^tm1(cre)Arbr^/J (PV‐Cre) heterozygous male mice (Stock No. 017320; The Jackson Laboratory, ME, USA) were group‐housed (two to four mice per cage) and handled for more than 1 week prior to the beginning of experiment. All mice were kept on a 12‐h reverse light/dark cycle (0800 lights off) with ad libitum access to food and water. All experiments took place during the dark phase. Animals were randomly assigned to each experimental group. All procedures regarding the use and handling of mice were conducted as approved by the Institutional Animal Care and Use Committee (IACUC) of the Korea Institute of Science and Technology (KIST).

### 2.2 Nicotine

Mice were exposed to nicotine via subcutaneous implantation of osmotic mini‐pump (Model 1004; Alzet, CA, USA). Briefly, mice were anesthetized with isoflurane (4% in pure oxygen for 3 min for induction, and 1.5% for maintenance). Nicotine solution was prepared immediately before osmotic pump implantation. (−)‐Nicotine ditartrate (Tocris Bioscience, MO, USA) was dissolved in physiological saline, and pH was adjusted to 7.4. Concentration was adjusted to deliver free‐base nicotine at 24 mg/kg/day for 2 weeks as previously described.^23^, ^24^ An incision was made in the back of the mice, the nicotine solution‐filled osmotic pump was inserted subcutaneously, and the wound was closed with silk suture. After 2 weeks of implantation (chronic nicotine exposure), the osmotic pump was surgically removed under isoflurane anesthesia to induce spontaneous nicotine withdrawal. For behavior tests, the control group underwent sham surgery in which mice were implanted with a mini‐pump without nicotine solution.

Single‐unit recording and behavior tests were conducted over 6 weeks (Figures 2A, 4A, 5C, and S4). The schedule consisted of weekly recording or behavior test sessions. Data collected during the first 2 weeks before osmotic pump implantation were pooled and used as "Baseline" phase. Data collected during the next 2 weeks after osmotic pump implantation were used as "Nicotine exposure" (or "Exposure") phase. Data collected during the last 2 weeks after osmotic pump removal were used as "Nicotine withdrawal" (or "Withdrawal") phase.

### 2.3 In vivo tetrode single‐unit recording

Single units were recorded simultaneously from the right dorsal striatum (center of the tetrode bundle, anteroposterior [AP], +0.7 mm; mediolateral [ML], ±1.9 mm from the midline; and dorsoventral [DV], 2.0–2.6 mm ventral to dura) with four tetrodes. Tetrode was fabricated by twisting four strands of nichrome wire (RO800 0.0127 mm (0.0005″) 1/4 Hard PAC; Kanthal, FL, USA) together and heating to fuse the insulation. The tetrode tip was cut and gold plated to reduce impedance to 0.2–0.3 MΩ following a previous study.^25^ Final impedance was measured at 1 kHz (NanoZ; Neuralynx, MT, USA). Tetrodes were then mounted on the skull of mice using Harlan 4 drive (Neuralynx). Tetrodes were slightly lowered 1–2 days before each recording session. Unit signals were recorded in home cage for 15 min per session, and the last 5 min of the recording was used for analysis. Signals were amplified (RHD2132 16‐channel amplifier board with 16 unipolar inputs; Intan Technologies, CA, USA), bandpass filtered between 0.6 and 6 kHz, digitized at 30 kHz (RHD2000 USB interface board; Intan Technologies), and then stored on a personal computer.

**FIGURE 1:**
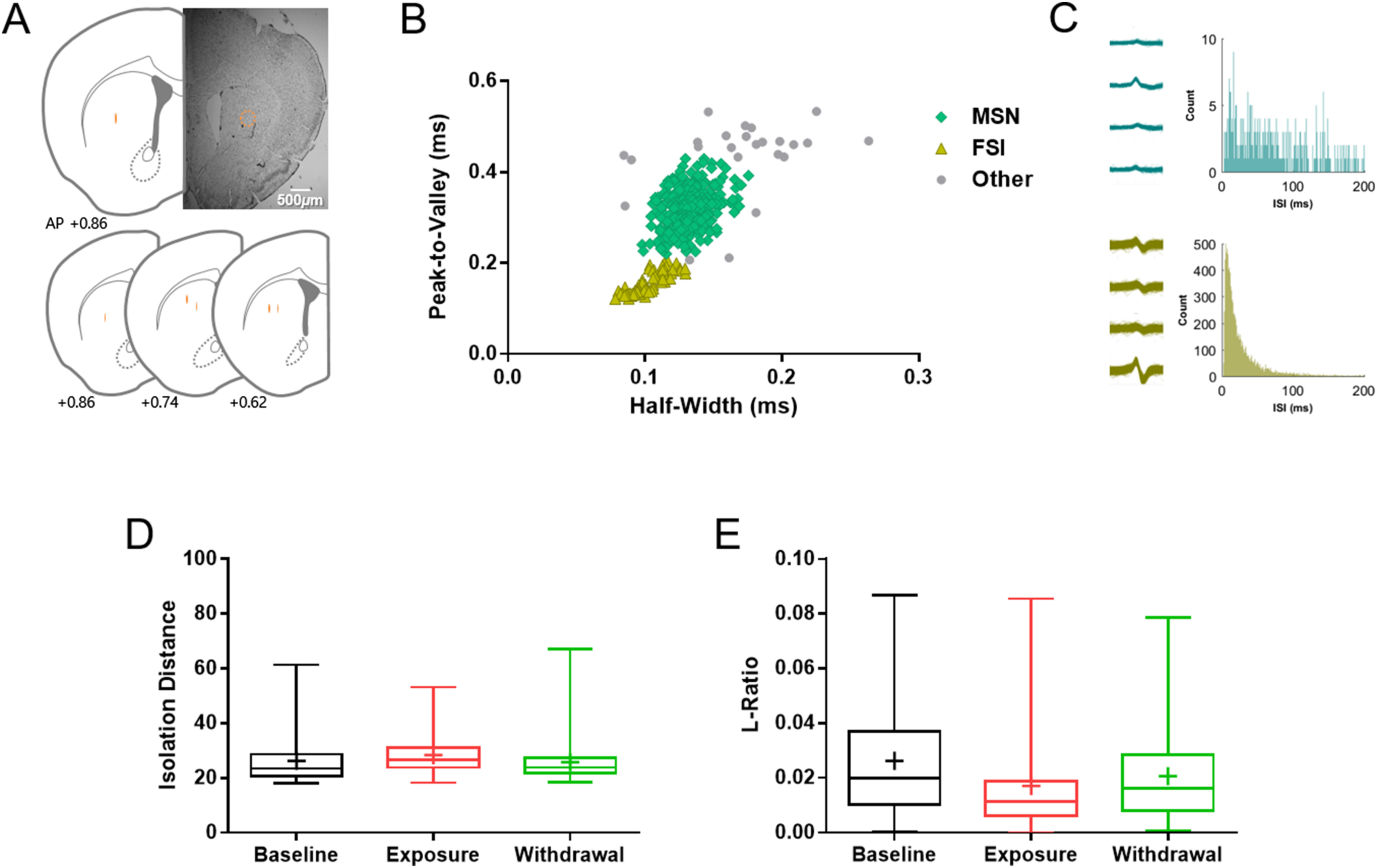
Validation of in vivo tetrode single‐unit recording. (A) Histological verification of the recording locations by Nissl staining. (B) Classification of recorded units as putative medium spiny neurons (MSNs; green; *n* = 309), fast‐spiking interneurons (FSIs; yellow; *n* = 64), or other neurons (gray; *n* = 24) by clustering according to wave properties. (C) Representative interspikeinterval (ISI) histogram of MSNs (above) and FSIs (below). (D) Isolation distance > 18 was used as a criterion for identifying units with acceptable isolation quality. Plus symbol indicates mean value. (E) L‐ratio < 0.09 was used as another criterion. Plus symbol indicates mean value

Single units were isolated via inspection of the 2D projections of waveform parameters and manually sorting the spikes in each cluster using custom software (WaveClus; R. Quian Quiroga, Caltech, CA, USA)^26^ with small modifications. The spike clusters were determined as a unit based on the waveform parameters, averaged spike waveform, autocorrelogram, and interspikeinterval histogram. The identified units were classified based on mean firing rate, spike half‐width, and peak‐to‐valley as in previous studies.^17^, ^27^ Those units with mean firing rate < 6.5 Hz, half‐width 0.1–0.16 ms, and peak‐to‐valley 0.25–0.37 ms were classified as putative MSNs, and those with half‐width 0.075–0.13 ms and peak‐to‐valley 0.12–0.17 ms were classified as putative FSIs (Figure 1B). Only those clusters with no interspike interval < 1 ms (Figure 1C), isolation distance > 18 (Figure 1D), L‐ratio < 0.09 (Figure 1E),^28^ and firing rate > 0.2 Hz were included in the analysis.

**FIGURE 2:**
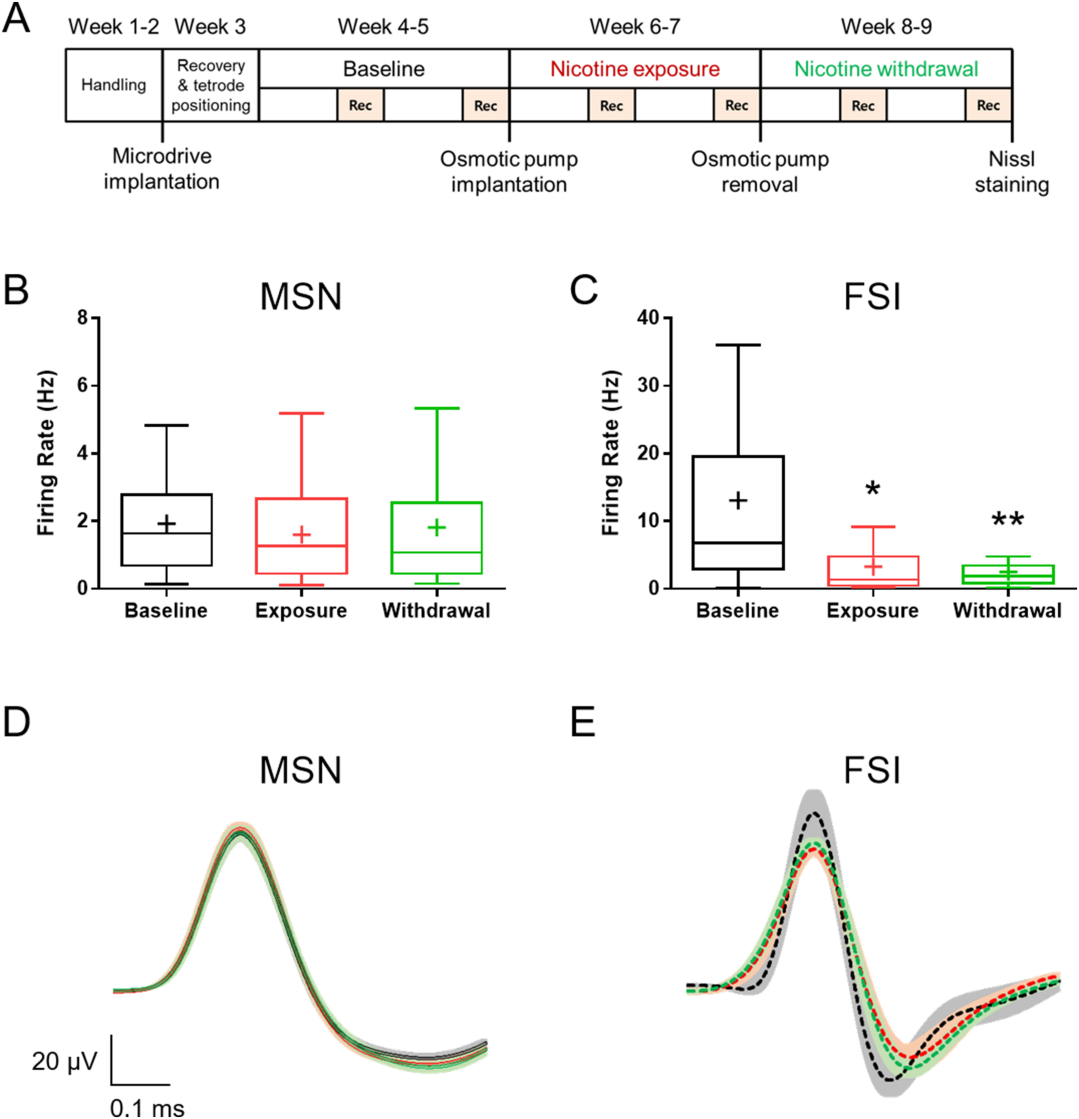
Chronic nicotine persistently suppresses the activity of striatal fast‐spiking interneurons. (A) Experimental schedule for 6 weeks of weekly longitudinal tetrode single‐unit recording in the dorsal striatum of mice. Rec indicates recording session. (B) Change in the firing rate of putative medium spiny neurons (MSNs) across three phases of recording sessions (Baseline, Exposure, Withdrawal; *n* = 85, 115, and 109, respectively). Plus symbol indicates mean value. Chronic nicotine treatment did not affect the activity of MSNs in vivo. (C) Change in the firing rate of putative fast‐spiking interneurons (FSIs) across three phases of recording sessions (Baseline, Exposure, Withdrawal; *n* = 21, 18, and 25, respectively). Chronic nicotine persistently reduced the activity of FSIs in vivo (^*^ *p* = 0.0212, ^**^ *p* = 0.0055). (D) Averaged spike waveform of the putative MSNs across three phases of recording sessions. Chronic nicotine does not notably alter MSN spike waveform. (E) Averaged spike waveform of the putative FSIs across three phases of recording sessions. Chronic nicotine alters the temporal characteristics of FSI spike waveform. Averaged spike waveforms at Baseline, Exposure, and Withdrawal are depicted in black, red, and green, respectively. Spike waveforms are aligned at the peak. Lines represent the mean spike waveform and shadows represent the standard error of the mean (SEM). Statistical comparison of the averages spike waveforms is depicted in Table 1

The firing rates and averaged spike waveform parameters of MSNs and FSIs were compared among three phases of recording sessions (Baseline vs. Exposure vs. Withdrawal) (Figure 2A). As depicted in Figure 2A, the units isolated from two recording sessions were pooled to represent each phase.

Spike waveforms were drawn by averaging the spike traces of units identified as either MSNs or FSIs across the three phases of recording sessions. For spike waveform analysis, we analyzed half‐width, peak‐to‐valley, spike amplitude, and amplitude ratio as in previous studies.^29^, ^30^ Briefly, half‐width is the time duration between half‐maximal amplitude points, peak‐to‐valley is the time duration between peak and valley, spike amplitude is the voltage difference between peak and valley, and amplitude ratio is the absolute value of the peak amplitude divided by valley amplitude.

In addition, the burst activity of MSNs were analyzed in parallel (Figure S1). Burst activity of MSNs is thought to be correlated with behavior‐relevant information including movement initiation and regulation.^31^, ^32^ A single burst event of MSNs was defined based on a previous report,^33^ with a small modification: A train of at least five spikes at an average firing rate of more than 20 Hz, and within which the interspike interval did not exceed 100 ms. The burst activity was analyzed through burst rate (the number of burst events per minute), intraburst firing rate (IBFR), and burst duration.

### 2.4 Local field potential

Local field potential (LFP) recording was performed as described previously,^34^ with small modifications. Briefly, the obtained data was bandpassed at 1–120 Hz and analyzed by Matlab (Mathworks; Natick, MA, USA). Thirty epochs (2 s each) of field potentials were randomly selected for analysis. For the power spectral density (PSD) analysis, the frequency ranges of delta (2–4 Hz), theta (4.5–8 Hz), alpha (8.5–12 Hz), beta (12.5–30 Hz), low gamma (30–55 Hz), and high gamma (60–85 Hz) were chosen. Sample entropy (SampEn) analysis was conducted following a previous report.^35^ SampEn measures the predictability of a signal using the conditional probability that two template sequences with similar points remain similar at the following points. In the computation of SampEn, the parameter *r* was used as the arbitrary threshold of similarity between two template sequences.

### 2.5 Nissl staining

After the single‐unit recording was completed, mice were deeply anesthetized by intraperitoneal administration of Avertin (2,2,2‐Tribromoethanol, 250 mg/20 ml/kg) (Sigma‐Aldrich, MO, USA), and microlesions were made on the recording sites by passing an electrolytic current (10 μA, 30 s, cathodal) through one channel of each tetrode. Subsequently, the whole brain was quickly isolated and freeze stored at −80°C. The frozen brain was coronally microdissected on a cryostat (CM3050S, Leica Microsystems, Wetzlar, Germany) to a thickness of 30 μm at −18°C. The brain sections were mounted on Superfrost Plus slide and air‐dried for ~10 min at room temperature. Then, the whole slide was sequentially incubated in xylene for 20 min, 100% ethanol for 5 min, 95% ethanol for 5 min, 70% ethanol for 5 min, distilled water for 5 s, cresyl violet (1.0 mg/ml, syringe filtered) for 10 min, distilled water for 2 min, 70% ethanol for 5 min, 95% ethanol for 5 min, 100% ethanol for 5 min, and xylene for 20 min. Finally, the slide was mounted with Permount mounting medium mixed with xylene at 1:1 ratio. The sections were imaged under light microscopy (Figure 1A).

### 2.6 Behavior

Mice were acclimated to the behavior test room for at least 30 min before the beginning of each experiment. The behavioral apparatus was wiped with 70% ethanol and distilled water before and between experimental sessions or blocks. The whole behavioral sessions were video recorded. As depicted in Figures 4A and 5C, the data obtained from two behavioral sessions were pooled to represent each phase (Baseline, Exposure, and Withdrawal).

#### 2.6.1 Open field

A white open field arena (40 * 40 * 40 cm inner dimension) was used. Luminosity on the floor of the open field was adjusted to ~5 lux. Mice were placed in the open field and allowed to freely explore the arena for 30 min. The total distance moved and time spent in the center zone were analyzed using EthoVision XT 11.5 (Noldus, Wageningen, Netherlands).

#### 2.6.2 Light–dark transition

The luminosity on the floor of the light chamber was adjusted to ~350 lux. Mice were placed in the dark chamber; then, the door to the light chamber was opened, and mice were allowed to freely explore the chambers for 10 min. The time spent in the light chamber was measured.

#### 2.6.3 Rotarod

Based on previous studies showing that the motor learning retention can be maintained for at least a week,^21^, ^22^ we established a sparse motor learning protocol with small modifications. A session consisted of three blocks with a 1‐h interblock interval. In the first block, mice were habituated on the rotarod with a fixed rate of 4 rpm for 5 min. In the second and third blocks, each block consisted of two consecutive trials. In a trial, mice were subjected to 4 rpm fixed‐speed training for 30 s and then were immediately subjected to accelerating rotarod (from 4 to 30 rpm, accelerating over 5 min). The better score out of two trials in the second block and the same in the third block were averaged to gain the latency to fall for the session.

To quantitatively assess the within‐group, sparse training‐dependent improvement in motor skill, we analyzed motor learning index based on previous studies,^36^, ^37^ with a small modification. Briefly put, the motor learning index is the motor performance of a mouse normalized by the average of the latencies to fall for an experimental group of mice at the Baseline phase. The motor learning index was defined as follows:

$$\text{Learning Index}=\frac{{\text{Performance}}_{\text{Session}}}{{\text{Mean Performance}}_{\text{Baseline}}}\;*100\%$$

in which the Mean Performance_Baseline_ indicates the mean latency to fall for an experimental group at the Baseline phase and Performance_Session_ indicates the latency to fall for each mouse in the experimental group at one session of a phase (Baseline, Exposure, or Withdrawal).

### 2.7 Adeno‐associated virus

pAAV‐hSyn‐DIO‐hM3D(Gq)‐mCherry (#44361; Addgene, MA, USA) and pAAV‐hSyn‐DIO‐mCherry (#50459; Addgene) were used for Cre‐dependent expression of an excitatory DREADD hM3Dq. Adeno‐associated virus (AAV) was packaged in the KIST Virus Facility (Seoul, Republic of Korea). Virus titer was 9.86 * 10^12^ GC/ml (Genome Copy/ml) for pAAV‐hSyn‐DIO‐mCherry and 3.70 * 10^12^ GC/ml for pAAV‐hSyn‐DIO‐hM3D(Gq)‐mCherry.

### 2.8 Intrastriatal microinfusion

For intrastriatal microinfusion of AAV, mice were anesthetized by intraperitoneal administration of ketamine‐xylazine mixture (120 and 8 mg/kg, respectively) and appropriately positioned in a stereotaxic frame (Kopf Instruments, CA, USA). Microinfusion was made into both hemispheres of the dorsal striatum at the following stereotaxic coordinates: AP, +0.7 mm; ML, ±1.9 mm from midline; and DV, −2.3 mm below the dura. The infusion was carried out by NE‐4000 Programmable 2 Channel Syringe Pump (New Era Pump Systems, NY, USA). The infusion volume was 0.2 μl, and the infusion rate was 0.1 μl/min. After infusion, the microneedle was held in position for 2 min.

### 2.9 DREADD‐mediated neuronal activation

Clozapine *N*‐oxide (CNO) dihydrochloride (Tocris) was dissolved in physiological saline to 0.03 mg/ml. For activation of the excitatory DREADD hM3Dq, the CNO solution was injected through intraperitoneal administration route at a concentration of 0.3 mg/10 ml/kg at 30 min prior to the beginning of each behavioral session or transcardial perfusion.

### 2.10 Immunohistochemistry

Mice were deeply anesthetized by intraperitoneal administration of Avertin (250 mg/20 ml/kg). Then, transcardial perfusion was performed with 1× PBS followed by 4% paraformaldehyde (PFA) in 1× PBS. The whole brain was isolated, post‐fixed in 4% PFA for overnight at 4°C, dehydrated with 30% sucrose until submersion, embedded in OCT compound, and stored at −80°C until cryosectioning. OCT‐embedded brains were coronally microdissected on a cryostat to a thickness of 40 μm at −20°C. Free‐floating sections were rinsed in 1× PBS followed by 0.3% Triton X‐100 in 1× PBS (PBST). Washed sections were blocked with 10% normal donkey serum (NDS) in 1× PBST for 1 h, then the sections were incubated with rabbit anti‐c‐Fos (1:2000) (sc‐52; Santa Cruz Biotechnology, CA, USA), mouse anti‐parvalbumin (1:1000) (MAB1572; Millipore, MA, USA), rabbit anti‐cleaved caspase‐3 (1:500) (9664S; Cell Signaling Technology, MA, USA), or rabbit anti‐DsRed (1:500) (#632496, Takara Bio Inc., Japan) for 2 days (anti‐c‐Fos) or overnight in 1× PBST with 1% NDS at 4°C. Subsequently, the sections were washed and incubated with appropriate secondary antibody for 2 h at room temperature. Finally, the sections were washed and mounted with VECTASHIELD HardSet Antifade Mounting Medium with DAPI (Vector Laboratories, CA, USA) according to the manufacturer's protocol. Images were taken with FluoView FV1000 (Olympus, Tokyo, Japan) and processed using FluoView software (Olympus); then, the images were visualized using Zen software (Carl Zeiss, Oberkochen, Germany) and ImageJ (National Institute of Health, Bethesda, MD, USA).

### 2.11 Experimental design and statistical analyses

Before proceeding to statistical comparisons of single‐unit spiking properties, Shapiro–Wilk normality test was conducted to analyze the skewness of distribution of each group (Table S1). For the within‐group comparison of firing rates (Figure 2), spike waveform parameters (Table 1), and burst activity of MSNs (Figure S1), Kruskal–Wallis nonparametric test followed by *Dunn* multiple comparison was applied because the sample distribution of spike properties was determined to be nonnormal (Table S1).

**TABLE 1:** Chronic nicotine alters the spike waveform properties of striatal fast‐spiking interneurons

Unit	Period	Half‐width (ms)	Peak‐to‐valley (ms)	Spike amplitude (μV)	Amplitude ratio
MSN	Baseline	0.1307 ± 0.0013	0.3132 ± 0.0045	90.02 ± 3.52	1.868 ± 0.038
	Exposure	0.1312 ± 0.0009	0.3181 ± 0.0039	93.82 ± 3.59	1.738 ± 0.026^*^
	Withdrawal	0.1300 ± 0.0008	0.3228 ± 0.0030	92.29 ± 3.67	1.726 ± 0.028^*^
FSI	Baseline	0.0849 ± 0.0045	0.1344 ± 0.0061	109.00 ± 13.14	1.552 ± 0.088
	Exposure	0.1082 ± 0.0024^***^	0.1636 ± 0.0035^**^	86.21 ± 3.42	1.707 ± 0.128
	Withdrawal	0.1084 ± 0.0027^***^	0.1624 ± 0.0032^**^	91.59 ± 3.67	1.570 ± 0.087

*Note*: Comparison of averaged spike waveform parameters in striatal putative striatal medium spiny neurons (MSN) (Baseline, Exposure, and Withdrawal; *n* = 85, 115, and 109, respectively) and putative striatal fast‐spiking interneurons (FSI) (Baseline, Exposure, and Withdrawal; *n* = 21, 18, and 25, respectively). Chronic nicotine slightly but significantly reduced the amplitude ratio of putative striatal MSN (^*^ *p* = 0.0413 for Baseline vs. Exposure, ^*^ *p* = 0.0233 for Baseline vs. Withdrawal). On the other hand, chronic nicotine induced a lasting increase in the half‐width and peak‐to‐valley of the averaged spike waveforms of putative FSIs (^***^ *p* = 0.0007 for Baseline vs. Exposure, ^***^ *p* = 0.0004 for Baseline vs. Withdrawal for half‐width; ^**^ *p* = 0.0011 for Baseline vs. Exposure, ^**^ *p* = 0.0029 for Baseline vs. Withdrawal for peak‐to‐valley).

**FIGURE 3:**
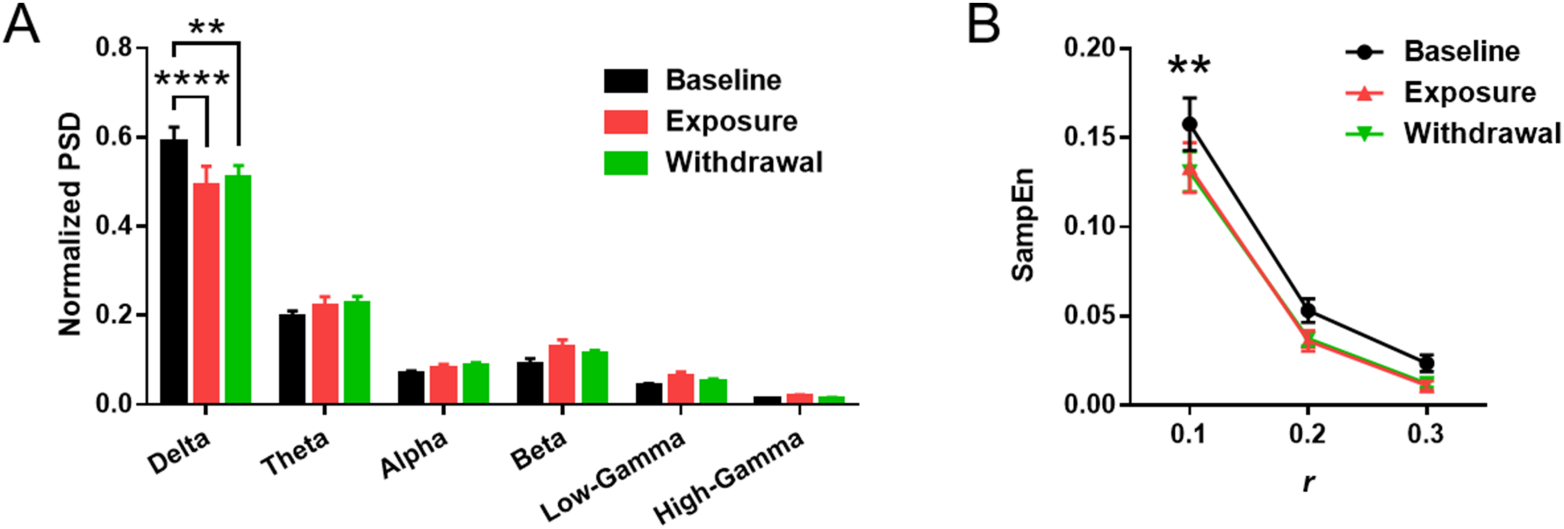
Chronic nicotine persistently diminishes striatal delta oscillation power and signal complexity. (A) Normalized power spectral density (PSD) of frequency bands across three phases of recording sessions (Baseline, Exposure, Withdrawal; *n* = 5 per group). Chronic nicotine persistently reduced the normalized power of delta oscillation (^**^ *p* = 0.0015, ^****^ *p* < 0.0001). (B) Sample entropy (SampEn) for measuring predictability of the signal across three phases of recording sessions. Chronic nicotine persistently reduced SampEn at *r* = 1, indicating increased predictability and reduced complexity of the signal (^**^ *p* = 0.0067 for Baseline vs. Exposure, ^**^ *p* = 0.0029 for Baseline vs. Withdrawal)

**FIGURE 4:**
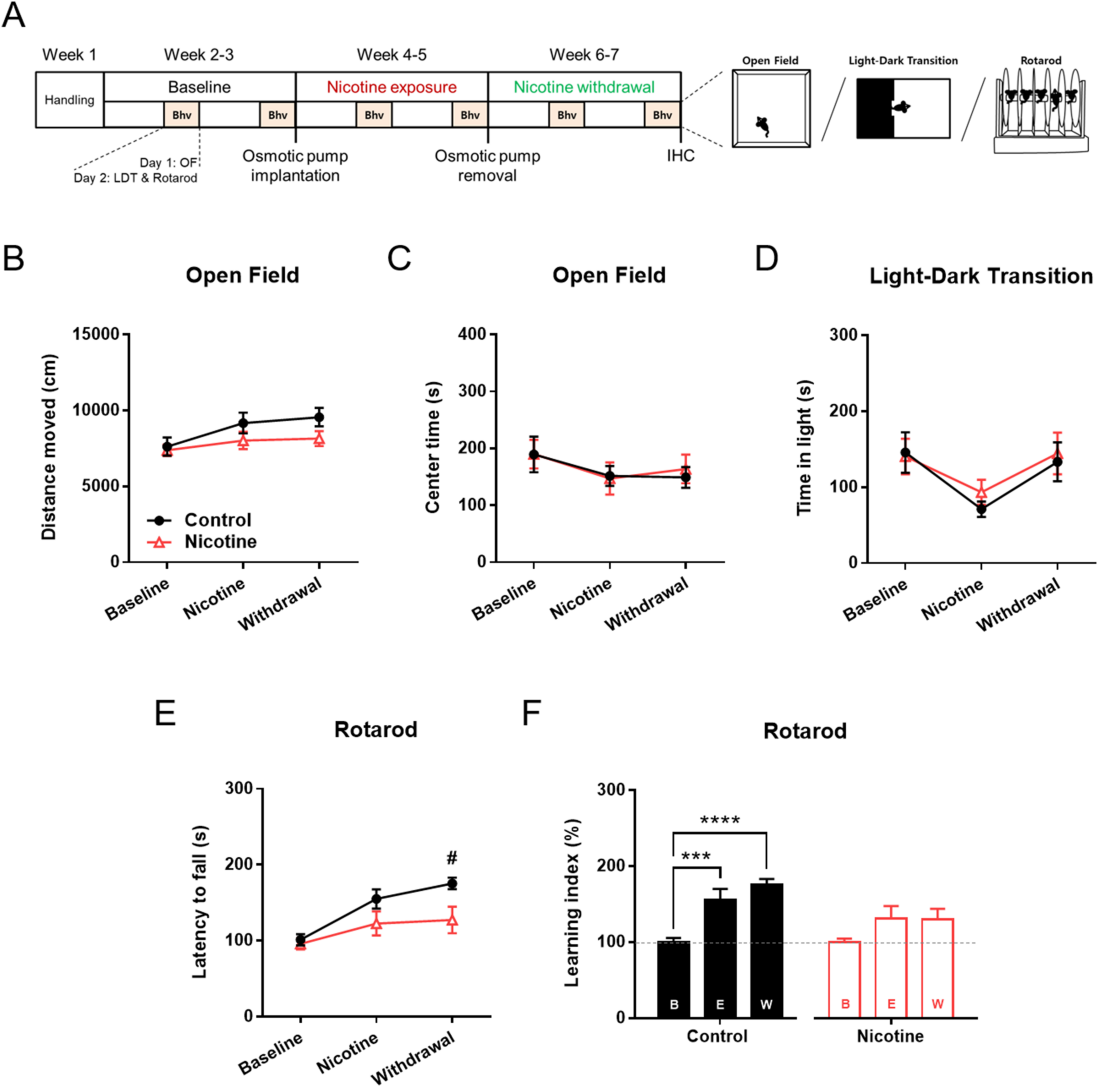
Chronic nicotine impairs sparse motor learning during withdrawal. (A) Experimental schedule for 6 weeks of weekly behavioral assay in mice. Bhv indicates behavioral session in which open field test (OF) was conducted at Day 1, and light–dark transition (LDT) followed by rotarod test was conducted at Day 2. Rotarod test was conducted 4 h after LDT. *n* = 14 per group. (B) Chronic nicotine did not significantly alter the distance moved in open field test. (C) Chronic nicotine did not alter the time spent in the center zone in open field test. (D) Chronic nicotine did not alter the time spent in the light chamber in light–dark transition test. (E) Chronic nicotine decreased the latency to fall in the rotarod during withdrawal phase (^#^ *p* = 0.0195). (F) Mice exhibited a gradual increase in motor learning index through the weekly sparse training regimen (^***^ *p* = 0.0006, ^****^ *p* < 0.0001), but chronic nicotine blocked the escalation of motor learning index. B indicates Baseline, E indicates Exposure, and W indicates Withdrawal phase

**FIGURE 5:**
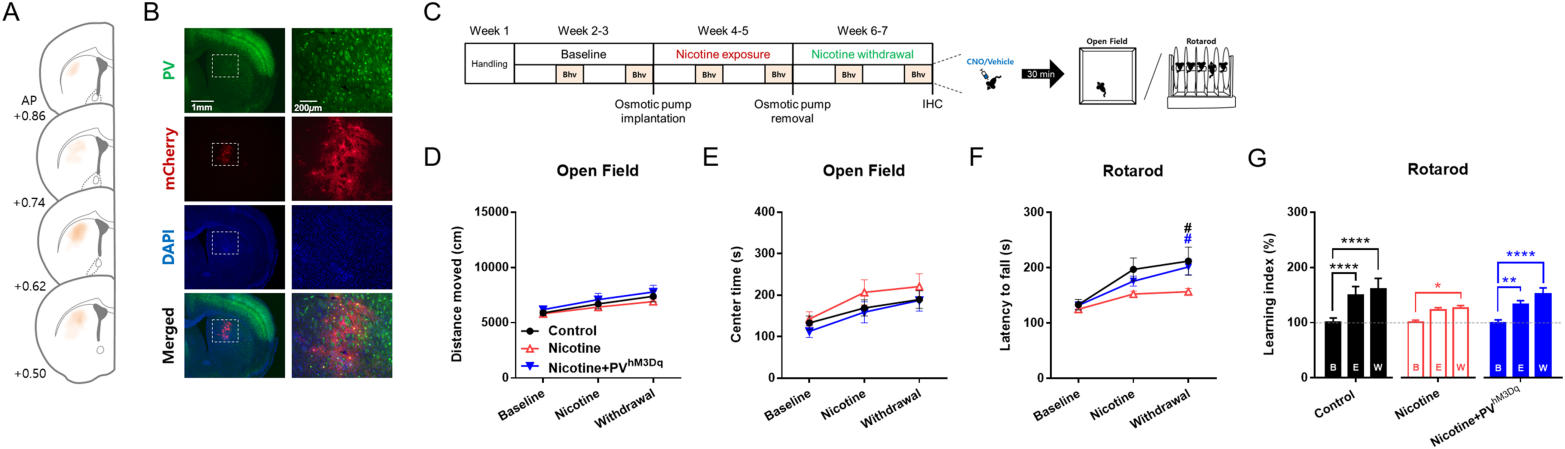
Chemogenetic activation of striatal parvalbumin interneurons reverses chronic nicotine‐induced deficit in sparse motor learning. (A) Adeno‐associated virus (AAV) microinjection site verified by mCherry signal. Distribution of mCherry signal (indicated by orange color) shows that AAV was spread throughout the central and lateral parts of the dorsal striatum. AP indicates anteroposterior in millimeter. (B) A representative image of parvalbumin (PV) signal merged with AAV‐driven mCherry signal spread throughout the dorsal striatum. (C) Experimental schedule for 6 weeks of weekly behavioral assay in mice. Bhv indicates behavioral session in which open field test (OF) was conducted at Day 1, and rotarod test was conducted at Day 2. Clozapine *N*‐oxide (CNO) or vehicle was injected at 30 min prior to the first block of each behavior session. *n* = 18, 18, and 16 for Control, Nicotine, and Nicotine + PV^hM3Dq^, respectively. (D) Neither chronic nicotine (Nicotine) nor chronic nicotine with activation of striatal PV interneurons (Nicotine + PV^hM3Dq^) significantly altered the distance moved in open field test. (E) Neither Nicotine group nor Nicotine + PV^hM3Dq^ group showed significant alteration of the time spent in the center zone in open field test. (F) Chronic nicotine decreased the latency to fall in the rotarod during withdrawal phase (^#^ *p* = 0.0107 for Control vs. Nicotine group), but activation of striatal PV interneurons reversed the latency to fall to the level comparable to that of Control group (^#^ *p* = 0.0420 for Nicotine vs. Nicotine + PV^hM3Dq^ group). (G) The gradual increase in motor learning index through the weekly sparse training regimen (Control group; ^****^ *p* < 0.0001) was attenuated by chronic nicotine (Nicotine group; ^*^ *p* = 0.0428), but activation of striatal PV interneurons reversed the motor learning index to the level comparable with that of Control group (Nicotine + PV^hM3Dq^ group; ^**^ *p* = 0.0088, ^****^ *p* < 0.0001). B indicates Baseline, E indicates Exposure, and W indicates Withdrawal phase

For the within‐group comparison of PSD (Figure 3A), SampEn (Figure 3B), and motor learning index (Figures 4F, 5G, and S4D), two‐way ANOVA followed by *Bonferroni* post hoc test was applied. For the between‐group comparison of distance moved, center time, latency to fall, and time in light (Figures 4, 5, and S4), two‐way ANOVA followed by *Holm–Sidak* post hoc test was applied. For comparison of PV + cells per striatal volume (Figure S2B) and c‐Fos + cells per PV + cells (Figure S3), Students *t* test was applied.

For all empirical tests, *p* < 0.05 was considered statistically significant, and significance was denoted as ^#^ *p* < 0.05, ^##^ *p* < 0.01, ^###^ *p* < 0.001, ^*^ *p* < 0.05, ^**^ *p* < 0.01, ^***^ *p* < 0.001, and ^****^ *p* < 0.0001. Results were displayed as Tukey box‐whiskers plot (Figures 2B,C and S1), mean ± standard error of the mean (SEM) (Figures 2D,E, 3, 4, 5, S2B, S3B, and S4), or box‐whiskers plot with whiskers from minimum to maximum (Figure 1D,E). In box‐whiskers plots, plus symbol indicates mean value.

All statistical analyses were conducted with Prism v6.0 (GraphPad, CA, USA).

## 3 RESULTS

### 3.1 Alteration of neural activity in the dorsal striatum after chronic nicotine

To first examine the impact of chronic nicotine on the striatal activity in vivo, we recorded the single‐unit activity of striatal neurons throughout 6 weeks of weekly longitudinal single‐unit recording sessions. Briefly, we implanted tetrodes onto the dorsal striatum of five mice (Figure 1A) and exposed mice to nicotine via osmotic mini‐pump. A total of 309 putative MSNs, 64 putative FSIs, and 24 other neurons were recorded (Figure 1). The majority of the analyzed units were MSNs (77.8%), and a small portion of units were FSIs (16.1%), the numbers of which are similar to striatal unit proportion reported in previous studies.^27^

To identify the long‐term effect of chronic nicotine on the activity of striatal MSNs and FSIs, we compared the change in firing rate across three phases of recording sessions (Baseline, Exposure, and Withdrawal) (Figure 2A). Quantitative comparison revealed that chronic nicotine did not change the firing rate and burst firing properties of MSNs across all phases (Figures 2B and S1; *n* = 85, 115, and 109 for Baseline, Exposure, and Withdrawal, respectively) but significantly and persistently diminished the firing rate of FSIs during both nicotine exposure and withdrawal phases (Figure 2C; *n* = 21, 18, and 25 for Baseline, Exposure, and Withdrawal, respectively; *H*(2) = 10.85; ^*^ *p* = 0.0212 for Baseline vs. Exposure, ^**^ *p* = 0.0055 for Baseline vs. Withdrawal).

To subsequently identify whether the impact of chronic nicotine extends to the intrinsic properties of striatal neurons, we analyzed spike waveform parameters of striatal MSNs and FSIs across three phases of recording sessions. Through visual inspection, we noticed that the averaged spike waveforms of MSNs display a subtle decrease in valley amplitude during both nicotine exposure and withdrawal (Figure 2D). Meanwhile, we also found that the spike waveforms of FSIs display broadened half‐width, delayed onset of valley, and concomitantly decreased amplitudes of peak and valley during both nicotine exposure and withdrawal (Figure 2E). In accordance, statistical analysis of the spike waveform parameters (Table 1) revealed that chronic nicotine subtly decreased the amplitude ratio of the MSNs (*H*(2) = 8.400; ^*^ *p* = 0.0413 for Baseline vs. Exposure, ^*^ *p* = 0.0233 for Baseline vs. Withdrawal), whereas substantially broadened the half‐width (*H*(2) = 17.33; ^***^ *p* = 0.0007 for Baseline vs. Exposure, ^***^ *p* = 0.0004 for Baseline vs. Withdrawal) and peak‐to‐valley (*H*(2) = 14.49; ^**^ *p* = 0.0011 for Baseline vs. Exposure, ^**^ *p* = 0.0029 for Baseline vs. Withdrawal) of the FSIs.

Previous studies have well‐established that the striatal FSIs are marked by parvalbumin immunoreactivity.^16^, ^17^ To exclude the possibility that chronic nicotine is reducing the firing of striatal FSIs via induction of neuronal cell death, we conducted an immunohistochemical analysis of cleaved caspase‐3 (Casp‐3), one of the most widely used neuronal apoptosis marker.^38^, ^39^ We confirmed that chronic nicotine withdrawal did not lead to induction of cleaved caspase‐3 and neuronal cell death in the parvalbumin interneurons (Figure S2A), and that chronic nicotine withdrawal did not change the number of parvalbumin interneurons in the dorsal striatum (Figure S2B; *n* = 4 per group).

Thereafter, we carried out LFP analyses to identify the effect of chronic nicotine on striatal field activity. LFP power analysis revealed that chronic nicotine exposure and withdrawal reduced the normalized PSD of delta oscillation (Figure 3A; *F*(5,20) = 149.7; ^****^ *p* < 0.0001 for Baseline vs. Exposure, ^**^ *p* = 0.0015 for Baseline vs. Withdrawal) but did not affect the power of other frequency bands. In addition, chronic nicotine also reduced the sample entropy (SampEn) (Figure 3B; *F*(2,12) = 76.03; ^**^ *p* = 0.0067 for Baseline vs. Exposure, ^**^ *p* = 0.0029 for Baseline vs. Withdrawal at *r* = 0.1).

In overall, chronic nicotine led to reduced activity of striatal fast‐spiking parvalbumin interneurons, but it did not necessarily lead to an increase in the activity of the striatal output neurons. The reduced firing of striatal fast‐spiking parvalbumin interneurons was accompanied by increased duration of half‐width and peak‐to‐valley. In addition, chronic nicotine modified the striatal slow‐wave activity and degraded the complexity of the striatal signal. More importantly, the chronic nicotine‐induced alterations of striatal activity were maintained throughout exposure to withdrawal. These results collectively indicate that chronic nicotine causes persistent disruption of the striatal neural activity in vivo.

### 3.2 Deficit in sparse motor learning during chronic nicotine withdrawal

Nicotine can influence motor learning in animals, and the dorsal striatum is critically involved in adaptive motor control. We thus hypothesized that the chronic nicotine‐induced disruption of striatal neural activity would lead to motor learning deficit. However, previous studies have demonstrated that nicotine does not affect motor learning at daily training schedule.^7^, ^8^ Therefore, we adopted sparse motor learning with a weekly training regimen (Figure 4A) based on previous findings that sparse training can unveil subtle behavioral deficits^18^, ^19^, ^20^ and motor learning retention can be maintained for over a week.^21^, ^22^

First, we found that chronic nicotine did not significantly affect general locomotion and anxiety‐like behaviors in the open field and light–dark transition tests (Figure 4B–D). However, chronic nicotine withdrawal resulted in significant reduction of the latency to fall from rotarod (Figure 4E; *n* = 14 per group; *F*(1,26) = 4.662; ^#^ *p* = 0.0195 for Control vs. Nicotine during Withdrawal). Interestingly, sparse motor learning was impaired only during nicotine withdrawal and not during nicotine exposure, although there was a trend towards deficit (Figure 4E; *p* = 0.1229 for Control vs. Nicotine during Exposure).

Further analysis of the sparse motor skill learning through learning index revealed that motor performance gradually increased as sparse training regimen progressed (Control in Figure 4F; *F*(2,52) = 16.20; ^***^ *p* = 0.0006, ^****^ *p* < 0.0001), but the sparse learning‐dependent improvement in motor performance was hindered by chronic nicotine (Nicotine in Figure 4F).

In association with the data from single‐unit recording, we suspected that the nicotine‐induced reduction of activity in striatal fast‐spiking parvalbumin interneurons could be the antecedent of the deficit in sparse motor learning. Therefore, to assess the role of striatal fast‐spiking parvalbumin interneurons in nicotine‐induced motor learning deficit, we adopted the chemogenetic approach to reverse the nicotine‐induced reduction of firing in striatal fast‐spiking parvalbumin interneurons (Figure 5). To this end, we expressed the excitatory DREADD hM3Dq in the striatal parvalbumin interneurons of PV‐Cre mice (Figures 5A,B and S3) and performed behavioral experiments (Figure 5C). CNO was systemically injected into the mice expressing hM3Dq in the striatal parvalbumin interneurons (PV^hM3Dq^) at 30 min prior to the beginning of each behavioral experiment.

First, the distance moved or time spent in the center zone in the open field test were unaffected by chemogenetic activation of striatal parvalbumin interneurons in nicotine‐exposed PV‐Cre mice (Figure 5D,E). Next, CNO‐induced activation of striatal parvalbumin interneurons led to recovery of the latency to fall from rotarod during chronic nicotine withdrawal to the level comparable to that of Control PV‐Cre mice (Figure 5F; *n* = 18, 18, and 16 for Control, Nicotine, and Nicotine + PV^hM3Dq^, respectively; *F*(2,49) = 2.984; ^#^ *p* = 0.0107 for Control vs. Nicotine and ^#^ *p* = 0.0420 for Nicotine vs. Nicotine + PV^hM3Dq^).

In accordance, the motor learning index gradually and substantially increased through sparse motor learning in PV‐Cre mice (Control in Figure 5G; *F*(2,98) = 31.49; ^****^ *p* < 0.0001), but chronic nicotine led to only marginal increase in the learning index (Nicotine in Figure 5G; *p* = 0.0999 for Baseline vs. Nicotine and ^*^ *p* = 0.0428 for Baseline vs. Withdrawal). Also, the chronic nicotine‐induced reduction of motor learning index was reversed by CNO‐induced activation of striatal parvalbumin interneurons (Nicotine + PV^hM3Dq^ in Figure 5G; ^**^ *p* = 0.0088 and ^****^ *p* < 0.0001).

Lastly, previous studies have shown that CNO alone could affect behavior possibly through back‐conversion of CNO to clozapine and subsequent interaction between signaling from DREADD with signaling from endogenous receptors associated with clozapine.^40^ To reveal the potential off‐target effect of CNO and the potential interaction between CNO and nicotine, we conducted open field and rotarod tests with PV‐Cre mice that received CNO alone or in conjunction with chronic nicotine (Figure S4; *n* = 16, 14, and 16 for Control, CNO, and Nicotine + CNO, respectively). We found that neither CNO nor chronic nicotine with CNO affected the distance moved and time spent in the center zone in the open field test (Figure S4A,B). In addition, CNO alone did not affect sparse motor learning and did not alter the chronic nicotine‐dependent deficit in motor learning (Figure S4C; *F*(2,43) = 5.301; ^##^ *p* = 0.0096 for Control vs. Nicotine + CNO, ^###^ *p* = 0.0004 for CNO vs. Nicotine + CNO).

Finally, the motor learning index gradually increased irrespective of CNO treatment (CNO in Figure S4D; *F*(2,86) = 26.44; ^**^ *p* = 0.0088 for Baseline vs. Exposure, ^**^ *p* = 0.0066 for Exposure vs. Withdrawal, ^****^ *p* < 0.0001 for Baseline vs. Withdrawal) whereas the chronic nicotine‐dependent reduction in the learning index was unaffected by CNO (Nicotine + CNO in Figure S4D).

**FIGURE 6:**
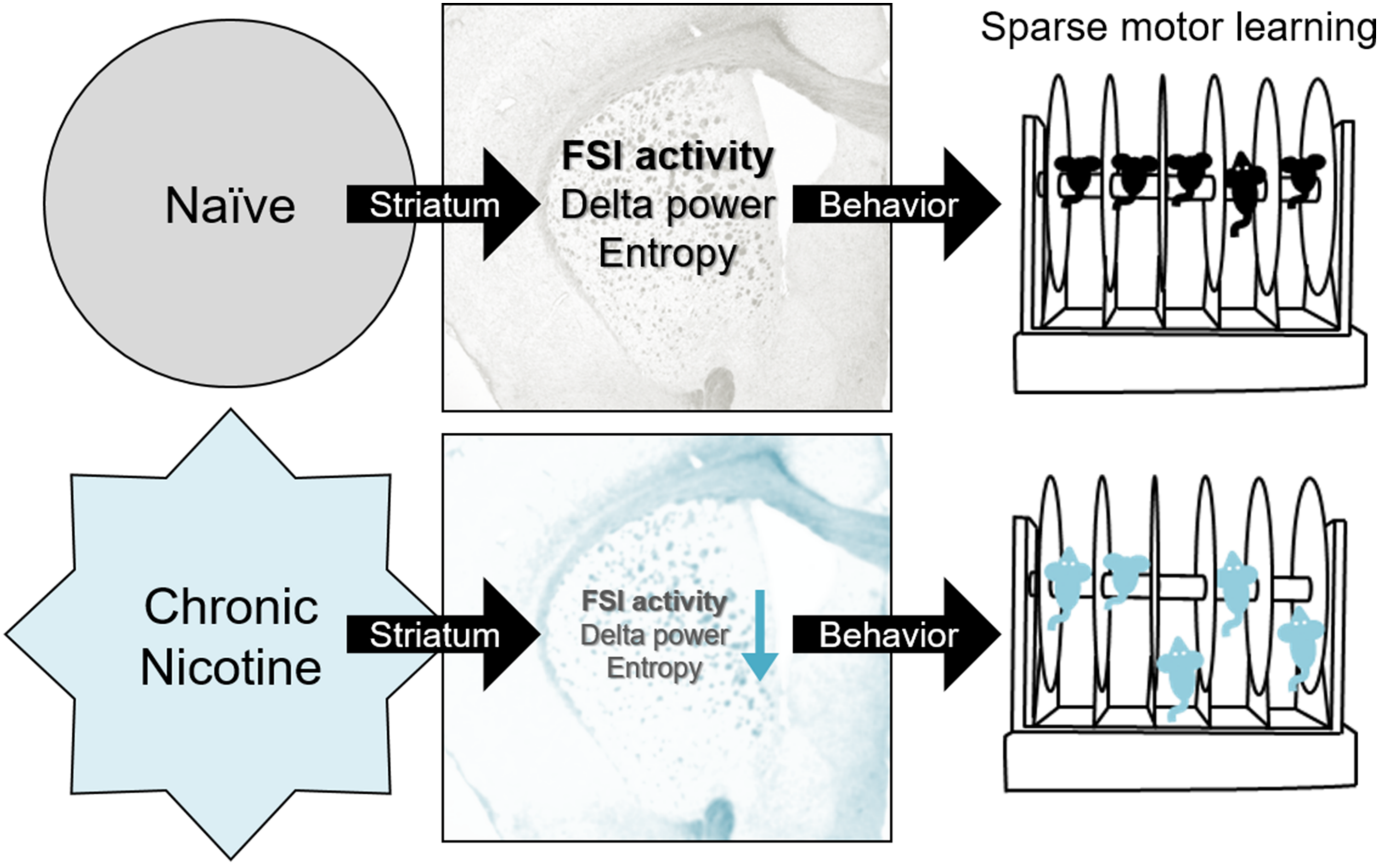
Schematic summary. Sparse motor learning requires the activity of striatal fast‐spiking parvalbumin interneurons (FSI). In the process, FSI activity may be correlated with striatal delta oscillation power and entropy. Chronic nicotine impairs striatal FSI firing, which is accompanied by reduced delta power and entropy. Moreover, chronic nicotine causes sparse motor learning impairment in mice. Chemogenetic reversal of nicotine‐induced deficit in striatal FSI firing rescues sparse motor learning, suggesting that the dorsal striatum is critical for motor learning and nicotine‐induced motor impairment

In sum, we discovered that chronic nicotine withdrawal impaired sparse motor learning and that striatal fast‐spiking parvalbumin interneurons mediate the nicotine‐induced deficit in sparse motor learning (Figure 6). These data collectively indicate that chronic nicotine causes subtle yet distinct impairment in motor behavior and that the striatal fast‐spiking parvalbumin interneurons play an important role in nicotine's influence over motor learning.

## 4 DISCUSSION

### 4.1 The effect of chronic nicotine on striatal activity in vivo

Across 6 weeks of longitudinal single‐unit recording, we found that chronic nicotine leads to a persistent and concurrent suppression of activity in the striatal fast‐spiking parvalbumin interneurons, striatal delta oscillation power, and field signal complexity. Here, the reduced striatal delta oscillation suggests impairment in striatum‐dependent behavior as previously suggested.^41^, ^42^ Also, reduced striatal delta power is consistent with the previous reports showing that striatal fast‐spiking parvalbumin interneurons are required for the generation of striatal delta oscillation in awake, behaving mice.^43^, ^44^ Moreover, reduction in entropy suggests simplified and atypical network dynamics in the dorsal striatum,^45^, ^46^ which is in accordance with the previous reports showing that FSIs expand the dynamic range of network responses.^47^, ^48^ These findings collectively suggest that chronic nicotine limits the neuronal and network activity in the dorsal striatum in vivo. However, the striatal FSIs as the origin of chronic nicotine‐dependent changes in striatal network activity should be experimentally verified in the future.

There are a number of possibilities that could link together chronic nicotine, FSIs, and the deficit in the striatal network activity. First, the nicotine‐induced reduction of firing in striatal FSIs could directly shape the striatal network responses. As previously found, nondesensitizing nicotinic acetylcholine receptors can directly depolarize striatal FSIs,^49^ and striatal FSIs can modulate both the striatal delta oscillation as well as the range of network responses.^43^, ^44^, ^47^, ^48^ Second, nicotine could work through other neurons to impact striatal FSIs and network activity. There are a variety of interneurons in the striatum,^50^, ^51^ each of which could be affected by nicotine^52^, ^53^ and can control striatal FSIs and striatal neural activity.^54^ In addition, the excitatory cortical/thalamic input to the dorsal striatum could be altered in response to chronic nicotine, thereby modulating both FSIs and striatal network response.^55^, ^57^ Moreover, the nicotine‐dependent alteration of dopaminergic action on striatal FSIs can also contribute to the overall process.^58^, ^59^ In the future, the existence and exact nature of causal relationship among chronic nicotine, striatal FSIs, and striatal network activity remain to be elucidated.

Here, we found that chronic nicotine reduces the activity of striatal fast‐spiking parvalbumin interneurons, which was accompanied by increased spike half‐width and peak‐to‐valley that gave rise to spike broadening. Previous studies have shown that spike broadening in the fast‐spiking neurons is largely correlated with reduced potassium currents from calcium‐activated BK channels and voltage‐gated Kv3 channels, and that potassium channel blocker can reduce the firing rate of fast‐spiking neurons.^60^ Interestingly, spike broadening has also been associated with increased calcium influx and enhanced neurotransmission,^61^, ^62^ which might compensate for the potential reduction in GABAergic neurotransmission from reduced activity of striatal FSIs and hence explain the unchanged firing of MSNs. In overall, these data imply that the potassium channels may underlie chronic nicotine‐dependent spike broadening as well as reduced activity of the striatal FSIs. However, the impact of nicotine on BK channels or Kv3 channels in the striatal FSIs remains unknown and should be investigated in the future.

The chronic nicotine‐mediated reduction of firing in striatal fast‐spiking parvalbumin interneurons contradicts the previous study demonstrating that acute activation of nicotinic receptors depolarizes the striatal FSIs ex vivo.^49^ A possible explanation to reconcile these findings is the differential effect of acute and chronic nicotine on neurophysiology and behavior. Previous studies have demonstrated that chronic nicotine can exert impact distinct from that of acute nicotine in many aspects, including synaptic plasticity, gene expression, and dopamine receptor signaling.^63^, ^64^, ^65^ In addition, a line of studies has revealed that learning and memory can be bidirectionally controlled by acute and chronic nicotine.^66^, ^67^, ^68^ These evidence suggest that acute and chronic nicotine can cause differential effects on the activity of striatal FSIs.

Also, a notable finding was that the reduced firing of striatal fast‐spiking parvalbumin interneurons did not accompany an increase in the firing rate of MSNs. The FSIs are an important modulator of a number of neurophysiological factors in the forebrain.^59^, ^69^ Briefly, the FSIs supply feedforward inhibition, sharpen spike timing, and expand the range of network responses.^70^, ^71^, ^72^ Interestingly, recent studies have shown that reduction in the firing rate of FSIs can lead to either elevated activity of MSNs^33^ or paradoxically decreased activity of MSNs through disinhibition of other striatal interneurons.^73^ However, the unchanged firing rate of MSNs in our study suggests that the chronic nicotine‐dependent reduction of activity in the striatal fast‐spiking parvalbumin interneurons may have interacted with any number of other possible effects induced by chronic nicotine, for example, changes in other striatal interneurons,^52^, ^53^ corticostriatal plasticity,^56^, ^74^ and striatal neurotransmitter release.^75^, ^76^, ^77^, ^78^, ^79^, ^80^ In addition, the chronic nicotine‐dependent spike broadening in the striatal fast‐spiking parvalbumin interneurons could also compensate for the reduced activity by enhancing GABAergic neurotransmission.^61^, ^62^ These views exemplify the complexity of nicotine's effect on striatal activity and preclude us from attributing the nicotine‐dependent neurophysiological alterations solely to fast‐spiking parvalbumin interneurons. Further studies are warranted to identify the roles of fast‐spiking parvalbumin interneurons on striatal activity in vivo under the context of nicotine.

Lastly, it is important to note that reduced firing of striatal FSIs was not accompanied by alteration of gamma oscillations. Previous studies have shown that striatal FSIs have strong relationship with the entrainment to gamma oscillations in the LFPs.^81^, ^82^ However, the sources of striatal gamma oscillations include volume conduction from piriform cortex, afferent synaptic inputs from other brain regions, and the activity of other striatal neurons. Complex interplay among the sources of striatal gamma oscillations and the result from this study suggest that change in FSIs' activity may not be the dominant factor in generation of striatal gamma oscillations.

### 4.2 The effect of chronic nicotine on behavior

Adopting 6 weeks of sparse motor learning paradigm, we found that chronic nicotine impairs motor learning in rotarod during withdrawal. This finding demonstrates that the motor learning deficit should be accounted for in the research of nicotine addiction, particularly during nicotine withdrawal. This finding is also important in light of the previous studies showing that chronic nicotine does not impact daily motor learning.^7^, ^8^ Sparse training is more sensitive to subtle deficits in behavior,^18^, ^19^, ^20^ which could explain the nicotine‐induced impairment in sparse motor learning but not in daily motor learning. In addition, our data supports the previous studies showing that the timescale of learning is a critical factor in behavioral assays.^83^, ^84^, ^85^, ^86^ Moreover, the dissociation of findings between daily and sparse motor learning suggests that continuous and sparse motor learning may be mediated by distinct neural mechanisms, which would be an interesting topic to explore in the future.

The most robust difference in motor skill performance was observed only during withdrawal. However, it is difficult to pinpoint whether the deficit in motor performance during nicotine withdrawal reflects a nicotine withdrawal‐specific motor deficit or an impairment in consolidation of learning during nicotine exposure. From careful examination of the data, a trend towards reduction in motor performance was observed during nicotine exposure, followed by a ceiling effect on performance during nicotine withdrawal. We suspected two possibilities: The motor deficit during nicotine withdrawal could be (1) an extension of subtle motor deficit from nicotine exposure or (2) a withdrawal‐specific deficit that was newly arisen after nicotine exposure. Supporting the former idea, the subtle reduction in motor performance observed during nicotine exposure implies that the impaired motor learning may have already been in place during exposure and lasted throughout withdrawal. However, the slope of learning was positive from baseline to exposure, whereas the slope of learning reached towards 0 from exposure to withdrawal. This suggests that motor learning was partially intact during nicotine exposure but was evidently absent during nicotine withdrawal. Collectively, we support the latter view that an exposure‐independent, withdrawal‐specific motor deficit caused the chronic nicotine‐dependent impairment in sparse motor learning.

In our study, anxiety‐like behavior was unaffected by chronic nicotine treatment. This finding is consistent with previous reports showing that chronic administration of nicotine does not affect anxiety‐like behavior in male mice.^87^, ^88^ However, it is also inconsistent with the previous reports showing that the ablation of FSIs leads to increased anxiety‐like behavior.^89^, ^90^ There are a number of possible explanations for this discrepancy. One possibility is the difference in the activity of striatal fast‐spiking parvalbumin interneurons within the striatal region. Although we found that chronic nicotine greatly reduced the activity of parvalbumin interneurons in the dorsal striatum, we did not examine the neural activity in all parts of the dorsal striatum. As shown in Figures 1A and 5A, tetrode positioning and AAV microinjection site were restricted to the central and lateral parts of the dorsal striatum. Because the dorsomedial striatum is more heavily involved in associative and emotional processing, anxiety‐like behavior could have been spared from chronic nicotine if the activity of fast‐spiking parvalbumin interneurons in the dorsomedial striatum were intact. Another possibility is due to the fast metabolic rate of nicotine in mice. Previous studies have demonstrated that mice exhibit a tremendously higher metabolic rate of nicotine than that of humans.^91^ This would vastly limit nicotine's duration of action in mice, which might underlie the challenge to observe behavioral phenotype without drug‐induced precipitation of the withdrawal signs as previously reported.^23^, ^92^ In this case, the anxiogenic level of open field and light–dark transition tests may not have been enough to unveil the subtle effect of spontaneous nicotine withdrawal on anxiety‐like behavior, whereas the high degree of task difficulty in the sparse motor learning could have interacted with the subtle effect of spontaneous nicotine withdrawal to reveal the nicotine‐induced motor learning deficit.

Lastly, the neurobehavioral difference between nicotine exposure and withdrawal was not observed in our study. Because the methods used in our study were limited in number and diversity, other neurophysiological or behavioral analyses could reveal the potential difference. In accordance, further research topics of interest include the longitudinal impact of chronic nicotine on other striatal interneurons/cell types and other dorsal striatum‐associated behaviors (e.g., sequence learning and habit formation). Moreover, the differential molecular alterations induced by nicotine exposure and withdrawal in the dorsal striatum would be another interesting topic to explore in the future.

### 4.3 Limitation

Only the trait phenotype was examined in the single‐unit recording experiment. The difficulty of single‐unit recording during rotarod test precluded us from examining the state phenotype, that is, neural activity during behavior. Nonetheless, information from the state phenotype is valuable for understanding the impact of chronic nicotine on behavior. Also, striatal cholinergic interneurons were not reliably identified and hence excluded from subsequent analysis. Previous studies found that striatal cholinergic interneurons are an important cellular substrate of motor function and dysfunction.^93^ Thus, it is possible that striatal cholinergic interneurons played a critical role in motor learning deficit induced by chronic nicotine. Further study with a more meticulous neurophysiological recording technique and a larger number of animals would allow both reliable measurement of neural activity on rotarod and thorough examination of the role of striatal cholinergic interneurons in nicotine‐induced motor learning deficit.

A main focus in our study was to reveal whether the striatal fast‐spiking parvalbumin interneurons are involved in nicotine‐induced motor deficit. In doing so, we have not explored other relevant points: (1) The neurophysiological consequences arising from chemogenetic activation of striatal fast‐spiking parvalbumin interneurons and (2) the intermediate mechanism by which the restoration of activity in striatal fast‐spiking parvalbumin interneurons reverses the behavioral effect of chronic nicotine. Without a thorough understanding of the impact of striatal fast‐spiking parvalbumin interneurons on nicotine‐associated behaviors and striatal activity, it would be premature to attribute the striatal fast‐spiking parvalbumin interneurons as the sole mediator of nicotine‐induced motor deficit.

Most importantly, the absence of relevant experimental groups in behavioral data limits us to make conservative interpretations. First, we did not use female animals in our study, precluding us from generalizing the data to the overall population. Although nicotine discrimination and seeking behaviors does not seem to differ between males and females,^94^, ^95^ mice do show sex differences in motor behaviors including the latency to fall on rotarod.^96^ Thus, the interaction between nicotine, motor skill, and sex may be an interesting topic to explore. Second, although we have demonstrated that CNO does not affect the chronic nicotine‐dependent impairment in sparse motor learning, the interactive effect of nicotine with CNO has not been thoroughly evaluated due to the limited scope of our study. The back‐conversion of CNO to clozapine could cause complex effects including its interaction with other pharmacological agents including nicotine.^40^ CNO alone does not affect rotarod learning,^97^, ^98^ and the dose used in our study (0.3 mg/kg) has been reported not to significantly affect baseline behaviors in rodents.^40^, ^99^ However, the potential interaction between CNO and nicotine should still be carefully inspected.

## 5 CONCLUSION

We employed a sparse motor learning paradigm to identify that chronic nicotine withdrawal leads to impairment in motor learning. We revealed through 6 weeks of longitudinal in vivo single‐unit recording and chemogenetic approach that the striatal fast‐spiking parvalbumin interneurons are a neural substrate of nicotine withdrawal‐induced motor learning deficit. Our data suggest that striatal fast‐spiking parvalbumin interneurons mediate the behavioral effect of nicotine and sparse motor learning, the notions of which may be extended to the research for nicotine dependence and movement disorders.

## DISCLOSURE/CONFLICT OF INTEREST

The authors declare no conflict of interest.

## AUTHORS CONTRIBUTION

H‐II supervised the project. BK designed and performed experiments. BK and H‐II analyzed the data and wrote the paper. All authors have critically reviewed content and approved final version submitted for publication.

## Supporting information


**Table S1.** Normality analysis for the spike data from tetrode single‐unit recording. Shapiro–Wilk normality test revealed that the spike data does not follow normal distribution. Therefore, the spike data was statistically analyzed with a nonparametric test method. MSN indicates medium spiny neurons, FSI indicates fast‐spiking interneurons, and IBFR indicates intraburst firing rate.

**Figure S1.** Chronic nicotine does not alter the burst firing properties of medium spiny neurons. (A) The burst rate, (B) intraburst firing rate (IBFR), and (C) burst duration of putative striatal medium spiny neurons (MSN) were unchanged by chronic nicotine treatment in vivo. *n* = 85, 115, and 109 for Baseline, Exposure, and Withdrawal, respectively.

**Figure S2.** Chronic nicotine withdrawal does not lead to loss of the striatal parvalbumin interneurons. (A) Cleaved caspase‐3 (Casp‐3), a marker for neuronal cell death, was not induced in neither the striatal parvalbumin (PV) interneurons nor any other cells after 2 weeks of withdrawal from chronic nicotine exposure. (B) Striatal PV + cell density (cells/mm3) was not affected by chronic nicotine withdrawal. *n* = 4/group.

**Figure S3.** hM3Dq‐mediated activation of striatal parvalbumin interneurons. (A) Immunohistochemical analysis of c‐Fos expression co‐localized to the mCherry (parvalbumin interneuron, PV) signal. (B) The excitatory DREADD hM3Dq‐mediated, clozapine N‐oxide (CNO)‐induced increase in the activation of striatal parvalbumin (PV) interneurons verified by c‐Fos expression co‐localized to the PV signal (c‐Fos+/PV+) (^*^ *p* = 0.0372). *n* = 5/group.

**Figure S4.** Clozapine N‐oxide does not affect sparse motor learning. (A) Neither clozapine N‐oxide (CNO group; *n* = 14) nor chronic nicotine with CNO (Nicotine+CNO group; *n* = 16) significantly altered the distance moved in open field test compared to the control (Control group; *n* = 16). (B) Neither CNO nor chronic nicotine affected the time spent in the center zone in open field test. (C) Chronic nicotine decreased the latency to fall in the rotarod during withdrawal phase irrespective of the CNO treatment (##*p* = 0.0096 for Control vs. Nicotine+CNO group, ###*p* = 0.0004 for CNO vs. Nicotine+CNO group). (D) Mice exhibited sparse training‐dependent increase in motor learning index (Control group; ****p < 0.0001) irrespective of the CNO treatment (CNO group; **p = 0.0088 for B vs. W, **p = 0.0066 for N vs. W, ****p < 0.0001 for B vs. W). On the other hand, chronic nicotine attenuated the gradual increase in motor learning index irrespective of the CNO treatment (Nicotine+CNO group). B indicates Baseline, E indicates Exposure, and W indicates Withdrawal phase.
