# Supplementary figures and images for: Chronic nicotine impairs sparse motor learning via striatal fast‐spiking parvalbumin interneurons
Source: Addict Biol. 2020 Aug 6;26(3):e12956. doi: 10.1111/adb.12956 (PMC8243919; doi:10.1111/adb.12956)

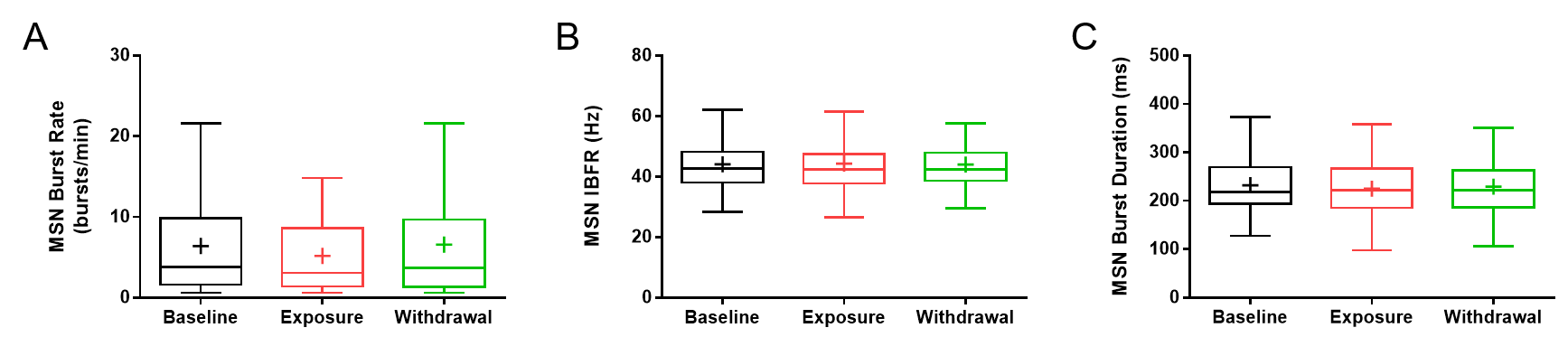

Supplement: Supplementary file 2 — Figure S1. Chronic nicotine does not alter the burst firing properties of medium spiny neurons. (A) The burst rate, (B) intraburst firing rate (IBFR), and (C) burst duration of putative striatal medium spiny neurons (MSN) were unchanged by chronic nicotine treatment in vivo. n = 85, 115, and 109 for Baseline, Exposure, and Withdrawal, respectively. [file ADB-26-e12956-s003.tif]

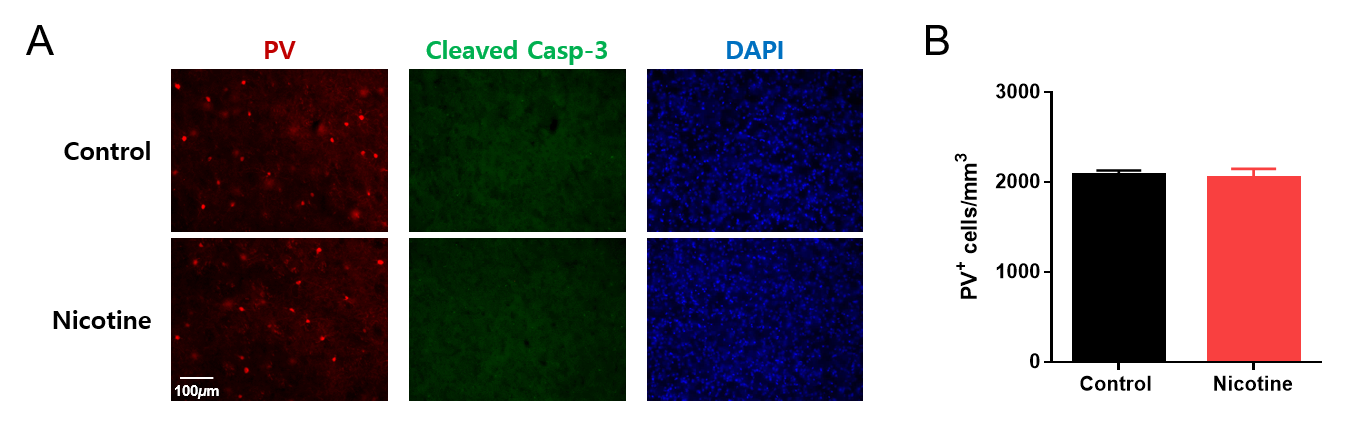

Supplement: Supplementary file 3 — Figure S2. Chronic nicotine withdrawal does not lead to loss of the striatal parvalbumin interneurons. (A) Cleaved caspase‐3 (Casp‐3), a marker for neuronal cell death, was not induced in neither the striatal parvalbumin (PV) interneurons nor any other cells after 2 weeks of withdrawal from chronic nicotine exposure. (B) Striatal PV + cell density (cells/mm3) was not affected by chronic nicotine withdrawal. n = 4/group. [file ADB-26-e12956-s005.tif]

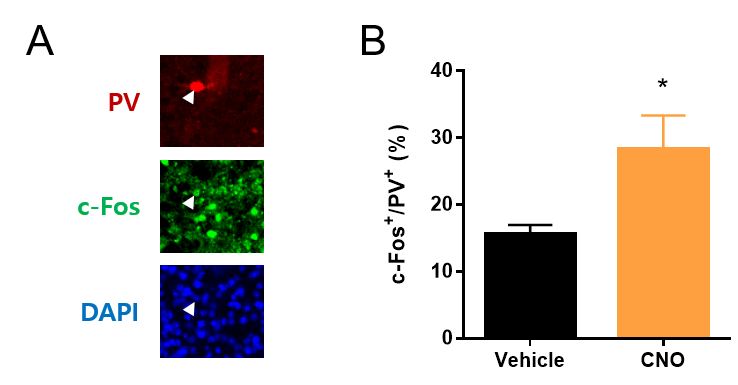

Supplement: Supplementary file 4 — Figure S3. hM3Dq‐mediated activation of striatal parvalbumin interneurons. (A) Immunohistochemical analysis of c‐Fos expression co‐localized to the mCherry (parvalbumin interneuron, PV) signal. (B) The excitatory DREADD hM3Dq‐mediated, clozapine N‐oxide (CNO)‐induced increase in the activation of striatal parvalbumin (PV) interneurons verified by c‐Fos expression co‐localized to the PV signal (c‐Fos+/PV+) (* p = 0.0372). n = 5/group. [file ADB-26-e12956-s004.tif]

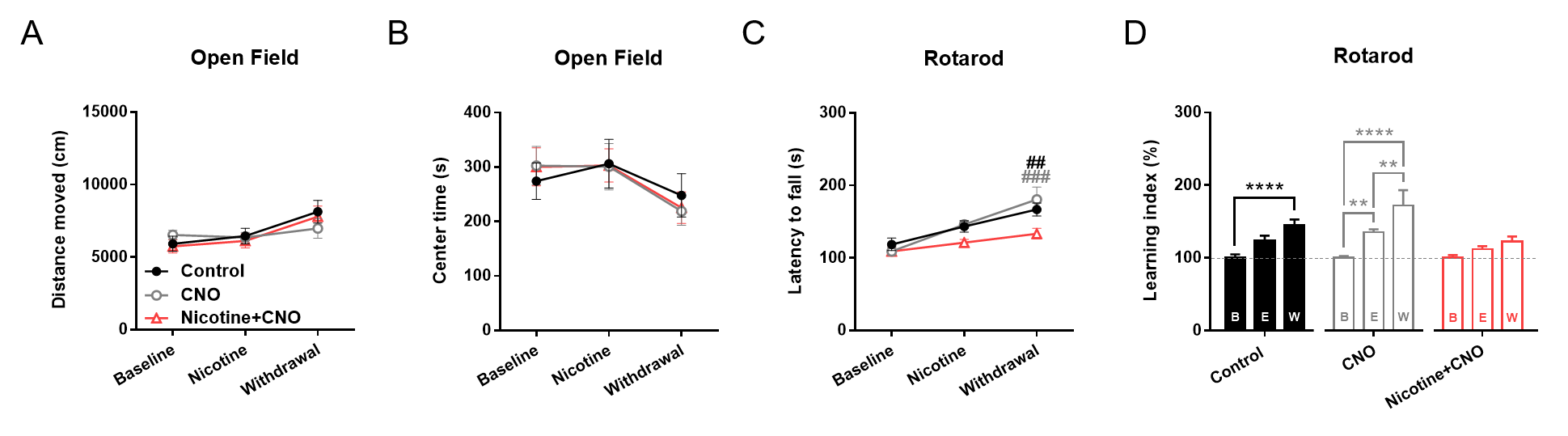

Supplement: Supplementary file 5 — Figure S4. Clozapine N‐oxide does not affect sparse motor learning. (A) Neither clozapine N‐oxide (CNO group; n = 14) nor chronic nicotine with CNO (Nicotine+CNO group; n = 16) significantly altered the distance moved in open field test compared to the control (Control group; n = 16). (B) Neither CNO nor chronic nicotine affected the time spent in the center zone in open field test. (C) Chronic nicotine decreased the latency to fall in the rotarod during withdrawal phase irrespective of the CNO treatment (##p = 0.0096 for Control vs. Nicotine+CNO group, ###p = 0.0004 for CNO vs. Nicotine+CNO group). (D) Mice exhibited sparse training‐dependent increase in motor learning index (Control group; ****p < 0.0001) irrespective of the CNO treatment (CNO group; **p = 0.0088 for B vs. W, **p = 0.0066 for N vs. W, ****p < 0.0001 for B vs. W). On the other hand, chronic nicotine attenuated the gradual increase in motor learning index irrespective of the CNO treatment (Nicotine+CNO group). B indicates Baseline, E indicates Exposure, and W indicates Withdrawal phase. [file ADB-26-e12956-s001.tif]
